# Selective diet induced nutritional optic neuropathy in developmentally normal children

**DOI:** 10.1016/j.ajoc.2024.102234

**Published:** 2024-12-10

**Authors:** Rhea W. Teng, Gena Heidary, Ryan A. Gise

**Affiliations:** a Department of Ophthalmology, Boston Children's Hospital, 300 Longwood Ave, Fegan 4, Boston, MA, 02115, USA; b Department of Ophthalmology, Harvard Medical School, 25 Shattuck Street, Boston, MA, 02115, USA

**Keywords:** ARFID, Avoidant/restrictive food intake disorder, Nutrient deficiency, Optic nerve atrophy, Optic neuropathy, Vitamin deficiency

## Abstract

**Purpose:**

Nutritional deficiencies in developed countries are a rare but potentially intervenable cause of optic neuropathy in pediatric populations. To date, much of the literature on nutritional optic neuropathy has focused on children with developmental delay, however, a growing body of evidence supports other underreported risk factors.

**Observations:**

We describe three pediatric patients with normal neurodevelopment, who presented with decreased vision and were subsequently found to have optic neuropathy attributed to vitamin deficiencies, predominantly vitamin B12. We review previous literature on nutritional optic neuropathy in pediatric patients, revealing that most published cases were associated with autism (17/25, 68 %).

**Conclusions and importance:**

An increasing number of cases, including our own, describe patients without autism who develop nutritional optic neuropathy due to restricted diets related to traumatic food-related events, multiple food allergies, or from an unknown cause. Altogether, our findings highlight the importance of a thorough diet and allergy review in pediatric patients with optic atrophy.

## 1 Introduction

Nutritional optic neuropathy is a rare, but potentially under-reported, cause of progressive vision loss, most commonly seen in developing countries.^1^ Visual loss is typically bilateral, subacute, progressive, relatively symmetric, and painless.^1^ A number of different vitamins, amino acids, and minerals have been implicated in the pathogenesis of nutritional optic neuropathy, particularly vitamins A, B1, B2, B6, B9, B12, homocysteine, methionine, and copper.^1^ In terms of pathogenesis, it is thought that lack of the aforementioned B vitamins and amino acids leads to disrupted mitochondrial oxidative phosphorylation, as well as subsequent energy depletion and free radical accumulation.^2^ Vitamin A deficiency, often recognized for worsening of retinal function and nyctalopia, has also been observed to cause abundant osteoid production in the optic canals, leading to compressive optic neuropathy in certain cases.^3^^,^^4^

Notably, risk factors for nutritional optic neuropathy vary greatly based on age. In adult patients, bariatric surgery, vegan diet, and alcohol use are common risk factors for nutritional deficiency.^1^ Comparatively, autism and its associated food selectivity seem to be the most commonly described risk factor in pediatric patients.^5^ Given the rising prevalence of disordered eating in pediatric patients, particularly after the coronavirus disease 2019 (COVID-19) pandemic, however, greater consideration of non-developmental risk factors is imperative.^6^^,^^7^

## 2 Methods

We present three pediatric patients with normal development who were evaluated by the Boston Children's Hospital neuro-ophthalmology department and found to have nutritional optic neuropathy (Table 1). Clinical data including age, sex, ophthalmic exam findings, clinical course, and laboratory and radiologic data were collected from the electronic medical record.

**Table 1:** Summary of each patient's ophthalmic exam findings and clinical course.

Case	Age at presentation to BCH	Sex	Diet	Presenting BCVA^1^	Presenting color vision^2^	Presenting clinical exam features	Presenting fundus photos	Presenting Humphrey Visual Fields MD	Presenting RNFL average (μm)	Presenting GCL Volume (mm^3^)	Treatment	BCVA at latest follow-up^1^
1	15	M	White rice, chicken nuggets/chicken, juice, soda, and candy	20/50+1 OD; 20/60+1 OS	11/11 OD; 11/11 OS	Unremarkable anterior segment; posterior segment with mild temporal disc pallor OU	Temporal optic disc pallor with peripapillary atrophy	MD24-2: 2.20 dB P <5 % OD; −2.42 dB P<5 % OS	91 OD; 92 OS	0.96 OD; 0.92 OS	Nutritional supplements; Oral vitamin B complex, vitamin D, calcium, and iron supplementation; IV calcium and vitamin K repletion; cyproheptadine	20/40–2 (NI PH) OD; 20/50–2 (NI PH) OS after 5 months of follow-up
2	13	M	Bacon, French fries, chicken nuggets, chips, select fruits	20/25 (NI PH) OD; 20/60 (20/25–3 PH) OS	10/10 OD; 10/10 OS	Unremarkable anterior segment; posterior segment with disc pallor OU	N/A	MD24-2: 12.32 dB P<0.5 % OD; −10.10 dB P<0.5 % OS	78 OD; 88 OS	0.71 OD; 0.64 OS	IV vitamin B9 repletion; IM vitamin B12 repletion; Oral multivitamin, vitamin B9, vitamin B12, vitamin A, vitamin D, and calcium supplementation	20/20–3 OD; 20/20–2 OS after 29 months of follow-up
3	15	M	Oatmeal, hash browns, waffles, rice, sweet potatoes, beans, broccoli, corn, apples, juice, oat milk, turkey bacon, rare chicken	20/60–1 OD (20/50–2 PH); 20/50–1 (20/40–2 PH)	8/8 OD; 8/8 OS	Unremarkable anterior and posterior segment OU	N/A	MD24-2: −1.80 dB P<10 % OD; −1.20 dB (low test reliability) OS	121 OD; 119 OS	0.82 OD, 0.83 OS	Oral vitamin B12 and vitamin D supplementation	20/60 (20/60+2 PH) OD; 20/70+2 (NI PH) OS after 27 months of follow-up

BCH, Boston Children's Hospital; BCVA, best corrected visual acuity; GCL, ganglion cell layer; IM, intramuscular; IV, intravenous; MD, mean deviation; NI, no improvement; N/A, not available; OD, right eye; OS, left eye; PH, pin hole; RNFL, retinal nerve fiber layer. ^1^Uncorrected visual acuity or VA with PH presented if BCVA not assessed. ^2^Color vision performed with Ishihara test.

A literature review was performed on PubMed from February 2024 to July 2024 without date restriction, using search terms *nutritional optic neuropathy* and *optic neuropathy* crossed with *autism, Avoidant/Restrictive Food Intake Disorder (ARFID), selective eating, restrictive eating, picky eating, food faddism, choosy eating, chronic food refusal, food neophobia, perseverant eating, food avoidance emotional disorder, functional dysphagia, pervasive refusal syndrome, food aversion.* Articles were limited to the English language. Age, sex, nutritional deficiencies, treatments, and visual outcomes were recorded. The study was exempt from approval from the Boston Children's Hospital Institutional Review Board because it included less than five patients.

## 3 Results

### 3.1 Case 1

**Fig. 1:**
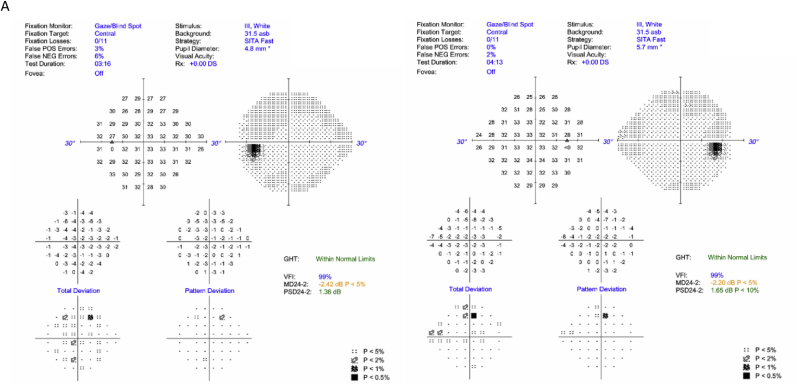

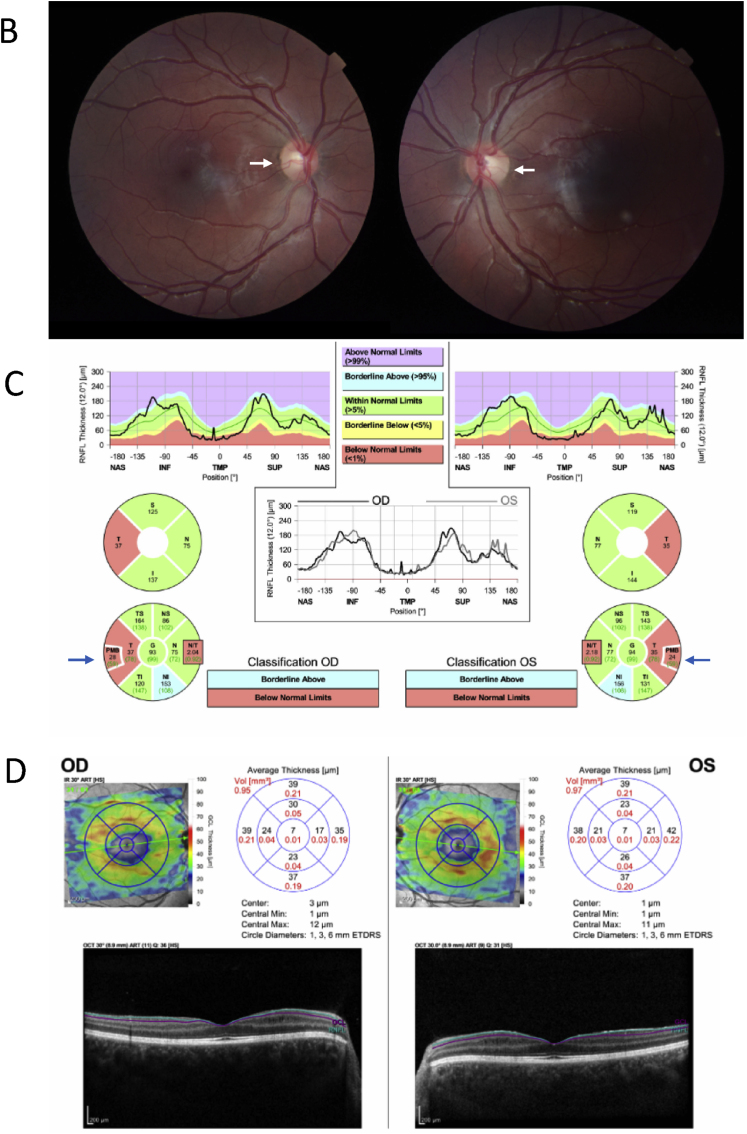
Case 1: 15-year-old male with one year of progressively blurry vision. A. Humphrey automated perimetry in each eye reliable and full at presentation. B. Fundus photos showing mild temporal disc pallor in both eyes (white arrows). C. Optical coherence tomography (OCT)-retinal nerve fiber layer (RNFL) from presentation demonstrating a borderline normal for age average RNFL with marked thinning of the papillomacular bundle in both eyes (blue arrows). D. OCT-ganglion cell layer (GCL) confirms generalized thinning and early optic atrophy in both eyes. (For interpretation of the references to color in this figure legend, the reader is referred to the Web version of this article.)

A 15-year-old male was referred to the neuro-ophthalmology department for evaluation of one year of increasingly blurry vision with best corrected visual acuity (BCVA) of 20/50 + 1 in the right eye (OD) and 20/60–1 in the left eye (OS). Past developmental and medical history were unremarkable. There was no family history of progressive vision loss. Allergies to cow's milk and eggs were previously recorded. On the initial exam, color vision was full. Humphrey automated perimetry was normal in each eye (Fig. 1A). On dilated fundus exam, mild temporal disc pallor was noted bilaterally (Fig. 1B). Optical coherence tomography (OCT)-retinal nerve fiber layer (RNFL) thickness measurements showed normal global thickness but temporal thinning bilaterally (Fig. 1C). OCT-ganglion cell layer (GCL) volume demonstrated generalized reduction compared to institutionally derived pediatric norms (Fig. 1D). Further history taking revealed that the patient was also allergic to seafood, wheat, tree nuts, sesame, and shellfish. Magnetic resonance imaging (MRI) of the brain and orbits without contrast was normal. Erythrocyte sedimentation rate and serology for syphilis, neuromyelitis optica (NMO), and myelin oligodendrocyte glycoprotein (MOG) antibodies were all normal/negative. Laboratory work-up was notable for low serum B6 (11.9 pmol/L; normal range 20.0–125.0 pmol/L) and B12 (140 pg/mL; normal range 211–911 pg/mL). In the setting of these vitamin deficiencies, the patient was diagnosed with nutritional optic neuropathy and referred to gastroenterology.

At gastroenterology, the patient shared that his diet consisted of white rice, chicken, juice, soda, and candy. He was diagnosed with ARFID, a new *Diagnostic and Statistical Manual of Mental Disorders, Fifth Edition* (DSM-5) defined condition of restrictive eating without body image disturbances or attribution to a concurrent medical condition or mental disorder, and started on a multivitamin with vitamin B complex and nutritional supplement drinks.^8^ Expanded labs showed significant hypocalcemia (7.3 mg/dL) and multiple micronutrient deficiencies (Table 2). He was admitted emergently for hypocalcemia management. In the inpatient setting, the patient underwent intravenous calcium and vitamin K repletion and was subsequently initiated on oral calcium, vitamin D, iron supplementation, and cyproheptadine for appetite stimulation. At six-months neuro-ophthalmology follow-up, his visual acuity was stable without further RNFL, GCL, or visual field (VF) losses.

**Table 2:** Summary of each patient's presenting nutritional deficiencies.

Case	Vitamin A (Normal range 20–80 mcg/dL)	Vitamin B1 (Normal range 70–180 nmol/L)	Vitamin B6 (Normal range 20.0–125.0 pmol/L)	Vitamin B9 (Normal range 4.2–20 ng/mL)	Vitamin B12 (Normal range 211–911 pg/mL)	Methylmalonic acid (Normal range 0.0–0.8 mcmol/L)	Copper (Normal range 85–150 mg/dL)	Ceruloplasmin (Normal range 20–43 mg/dL)
1	N/A	87	11.9 (L)	14.3	140 (L)	0.3	69 (L)	N/A
2	<10.0 (L)	217 (H)	N/A	1.4 (L)	<150 (L)	N/A	61 (L)	17 (L)
3	37.3	98	27.0	5.2	<150 (L)	0.6	88	N/A

H, high levels; L, low levels; N/A, not available.

### 3.2 Case 2

A 13-year-old male presented for abdominal pain and fatigue and was found to be pancytopenic, with severe anemia necessitating admission to the intensive care unit. Past medical and developmental history were notable for low body weight (<first percentile). Laboratory workup for the patient's anemia demonstrated low serum B9 (1.4 ng/mL; normal range 4.2–20 ng/mL), B12 (<150 pg/mL; normal range 211–911 pg/mL), ceruloplasmin (17 mg/dL; normal range 20–43 mg/dL), copper (61 mcg/dL; normal range 85–150 mg/dL), and vitamin A (<10.0 mcg/dL; normal range 20–80 mcg/dL). His diet consisted predominantly of bacon, French fries, chicken nuggets, and chips. He was diagnosed with ARFID after excluding other malabsorptive diseases, and his anemia was attributed to severe malnutrition. He underwent intravenous B9, intramuscular B12, and oral vitamin A repletion, and was initiated on an oral multivitamin, calcium, vitamin D, vitamin B9, and vitamin B12, to be continued in the outpatient setting.

Although the patient denied visual complaints, ophthalmology was consulted to assess for any nutritional deficiency-related changes. No family history of ophthalmic disorders was noted. His visual acuity was 20/25 in each eye. Color vision was normal in both eyes. Humphrey automated perimetry showed a small cecocentral defect in each eye. He had an unremarkable anterior segment exam and bilateral optic disc pallor on dilated fundus exam. OCT showed bilateral, diffuse RNFL thickness and GCL volume loss. MRI of the brain and orbits without contrast was normal, however, he had evidence of subacute combined degeneration on spinal MRI. This was also felt to be related to his nutritional deficiencies. He was diagnosed with nutritional optic neuropathy. The patient continued to follow with gastroenterology outpatient and was continued on multivitamin, calcium, and vitamin D supplementation after normalization of his labs. After two years of follow-up, his visual acuity, RNFL, GCL, and cecocentral scotomas remained unchanged.

### 3.3 Case 3

A 15-year-old male was referred for seven months of worsening blurry vision. He had originally been seen by an outside ophthalmologist two months prior, who noted 20/200 vision bilaterally and referred him to neurology to rule out optic nerve disease. MRI of the brain and orbits without contrast and lumbar puncture were normal. Developmental history was unremarkable. Past medical history was notable for atopic dermatitis, allergic rhinitis, and allergic conjunctivitis. Allergies to fish, peanuts, cow's milk, eggs, all tree nuts, sesame, and shellfish were noted. Family history was non-contributory. He reported limited meat consumption, consisting only of turkey bacon every morning and rarely chicken. His best corrected visual acuity was 20/50–2 OD and 20/40–2 OS. Color vision was normal in both eyes. Humphrey automated perimetry revealed a small central scotoma in each eye. Anterior and poster segment exams were normal. On OCT, there was papillomacular bundle and temporal thinning of the RNFL with a normal global average bilaterally. Testing for syphilis, NMO, and MOG antibodies was negative. Laboratory workup revealed low B12 (<150 pg/mL; normal range 211–911 pg/mL), and he was diagnosed with nutritional optic neuropathy. Over two years of follow-up, the patient's reduced visual acuity, central scotomas, and RNFL thinning have remained stable in both eyes.

## 4 Literature review

A total of 25 cases of nutritional deficiency-related optic neuropathy have been reported thus far in pediatric patients (Table 3).^4^^,^^5^^,^^9^, ^10^, ^11^, ^12^, ^13^, ^14^, ^15^, ^16^, ^17^, ^18^, ^19^, ^20^, ^21^, ^22^, ^23^ Ages ranged from 5 to 18 years old. Twenty-two out of 25 patients (88%) were male. Seventeen out of 25 patients (68%) had diagnoses of autism, and 1/25 (4%) had global developmental delay at the time of presentation. Patients presented with a variety of vitamin and micronutrient deficiencies, most commonly vitamins A and B12. In individuals with low vitamin A, comorbid Bitot's spots (6/22) and corneal pathologies (9/22) such as edema, haze, keratinization, epithelial defects, and punctate epithelial erosions were common. Hyperostosis of the optic canal leading to compressive optic neuropathy was implicated in 9/22 cases with low vitamin A. Of the patients with normal neurodevelopment, malnutrition was attributed to restrictive eating patterns, loss of appetite in the setting of modafinil therapy for narcolepsy treatment, ARFID, or unexplained.^17^, ^18^, ^19^, ^20^, ^21^, ^22^, ^23^ Interestingly, three of the patients without autism or global development delay were noted to have specific events after which their eating patterns became more restricted, including weight-based bullying, adenoidectomy, and choking.^20^, ^21^, ^22^ Nutritional deficiencies were repleted using intravascular, intramuscular, and oral repletion of specific vitamins, multivitamins, nutritional supplements, and dietary diversification. Eighteen of the 20 cases in which visual acuity was reported showed some level of improvement after vitamin repletion.

**Table 3:** Literature review of pediatric patients with nutritional optic neuropathy.

Number	Author	Age	Sex	Nutritional deficiencies	Neurodevelopmental or Relevant Conditions	Presenting VA	Ophthalmic findings	Treatment	Outcome
1	Chiu et al.^6^	12	M	Vitamin A, vitamin B9, iron	Autism; history of EBV-associated optic neuropathy OD with 6/60 vision	Light perception OD; 1/60 OS	Optic nerve pallor OD; temporal optic nerve pallor OS; bilateral corneal and conjunctival keratinization	Topical retinoic acid, high-dose oral vitamin A supplementation, multivitamin, lubricating eye drops, surgical decompression of the optic canal OS	Light perception OD; 2/60 OS
2	Lin et al.^7^	9	M	Vitamin A	Autism	Not specified	Optic nerve atrophy OS > OD; bilateral nonhealing epithelial defects	Oral vitamin A supplementation, frequent lubrication	Not specified
3	McAbee et al.^8^	17	M	Vitamin A	Autism	Hand motion OD; hand motion OS	Bilateral moderately severe optic nerve atrophy; bilateral Bitot's spots, edema of corneal limbus, diffuse corneal haze	IM vitamin A, oral multivitamin, E028 Splash nutritional drink	Functional improvement
4	Godfrey et al.^3^	17	M	Vitamin A, vitamin B12	Autism; MOG antibody +1:20	Unable to assess	Bilateral optic neuropathy secondary to hyperostosis of the optic canal	Vitamin A, steroids, mycophenolate mofetil	Improvement in night vision, but worsening after prednisone weaned
5	Godfrey et al.^3^	17	M	Vitamin A, vitamin E, vitamin K	Autism	20/200 OD; 20/200 OS	Bilateral optic neuropathy secondary to hyperostosis of the optic canal	Vitamin A, vitamin B1, vitamin D, vitamin K, leucovorin	20/50 OD; 20/80 OS
6	Godfrey et al.^3^	5	M	Vitamin A, vitamin A, vitamin B12	Autism	20/200 OD; 20/200 OS	Bilateral optic neuropathy secondary to hyperostosis of the optic canal	Vitamin A, vitamin B12, vitamin E; G-tube placement	20/30 OD; 20/30 OS
7	Godfrey et al.^3^	16	M	Vitamin A	Autism	20/200 OD; motion only OS	Bilateral optic neuropathy secondary to hyperostosis of the optic canal	Vitamin A	20/100 OD; 20/100 OS
8	Godfrey et al.^3^	12	M	Vitamin A, vitamin B12	Autism	Motion only OD; motion only OS	Bilateral optic neuropathy secondary to hyperostosis of the optic canal	Vitamin A, vitamin B12	20/200 OD; 20/200 OS
9	Godfrey et al.^3^	9	M	Vitamin A	Autism	20/200 OD; motion only OS	Bilateral optic neuropathy secondary to hyperostosis of the optic canal	Vitamin A	20/70 OD; 20/200 OS
10	Pineles et al.^4^	6	M	Vitamin A, Vitamin B12	Autism	20/130 OD; 20/130 OS on Teller visual acuity	Bilateral optic nerve atrophy	IM vitamin B12	Functional improvement
11	Pineles et al.^4^	13	M	Vitamin B12	Autism	No fix, follow, or blink to threat OD; No fix, follow or blink to threat OS	Bilateral optic nerve atrophy	IM vitamin B12	Functional improvement
12	Pineles et al.^4^	7	M	Vitamin B12	Autism	No fix, follow, or blink to threat OD; No fix, follow or blink to threat OS	Bilateral optic nerve atrophy	IM vitamin B12	Functional improvement
13	Pereira et al.^9^	12	M	Vitamin A	Autism	20/300 OD; hand motion OS	Bilateral optic neuropathy secondary to hyperostosis of the optic canal; bilateral reduced dark adaptation electroretinogram	Oral vitamin A supplementation; acetazolamide for increased intracranial pressure with vitamin A treatment	20/250 OD; count fingers OS
14	Cheah et al.^10^	8	M	Vitamin A	Autism	2/60 PH OD; hand motion OS	Bilateral temporal optic disc pallor; bilateral reduced dark adaptation electroretinogram	IM vitamin A	2/60 PH OD; hand motion OS
15	Duignan et al.^11^	14	M	Vitamin A, borderline vitamin B12, vitamin D	Autism	20/25 OD; 20/25 OS	Bilateral mildly swollen optic disc; bilateral Bitot's spots; bilateral rod-predominant retinopathy	IV methylprednisolone, IV acyclovir, oral vitamin A supplementation, dietary diversification	“Vision normalizing”
16	Azmi et al.^12^	7	M	Vitamin A	Autism	Light perception OD; light perception OS	Bilateral pale optic discs; bilateral conjunctival xerosis, generalized punctate epithelial erosions	“Systemic vitamin A therapy”	Hand motion OD; 1/60 OS
17	Azmi et al.^12^	7	M	Vitamin A	Autism	6/12 OD; No light perception OS	Swollen optic disc OD; severe leukomalacia with perforated cornea OS	“Systemic vitamin A therapy”	Not specified
18	Farrell et al.^13^	12	M	Vitamin A	Global developmental delays, severe food aversions	20/400 OD; counting fingers at 2 feet OS	Bilateral optic nerve pallor; bilateral severely decreased tear film, conjunctival xerosis, diffuse punctate epithelial erosions, mild corneal haze	Oral vitamin A supplementation	20/80 OD; 20/400 OS
19	Zayed et al.^14^	17	M	Vitamin A, vitamin D, vitamin E	Restricted diet	6/6 OD; hand motion OS	Left optic neuropathy secondary to hyperostosis of the optic canal	Unspecified vitamin supplements, dietary diversification	6/6 OD; hand motion OS
20	Bailey et al.^15^	13	M	Vitamin A, vitamin B2 borderline vitamin B12, borderline vitamin C, vitamin E	Restricted diet	No light perception OD; 20/125 OS	Bilateral optic disc pallor; bilateral large Bitot's spots with marked keratinization and dryness	“Replacement of multivitamins,” including parenteral vitamin A, vitamin B12, and vitamin B1 supplementation	Light perception OD; 20/20 OS
21	O'Neill et al.^16^	15	F	Vitamin A, vitamin B12, vitamin D, copper, iron	Restrictive eating patterns	Light perception OD; 20/20 OS	Bilateral optic nerve atrophy secondary to hyperostosis of the optic canal OD > OS; Bitot's spots	Limited adherence to oral vitamin supplementation	Not specified
22	Mina et al.^17^	15	M	Vitamin A, vitamin B9, vitamin B12, vitamin D	Avoidant/Restrictive Food Intake Disorder	6/60 OD; no light perception OS	Bilateral optic disc pallor	Vitamin A and vitamin D stoss therapy, multivitamin (vitamin B12, vitamin B9, calcium)	Not specified
23	Chiarello et al.^18^	18	M	Vitamin B9, vitamin B12, phosphate	Avoidant/Restrictive Food Intake Disorder	20/600 OD; 20/600 OS	Bilateral severe optic disc edema	Intramuscular vitamin B12, oral vitamin B9, sertraline for food related anxiety	Not specified
24	Chia et al.^19^	12	F	Vitamin A, vitamin B9, vitamin B12, vitamin D, copper	Avoidant/Restrictive Food Intake Disorder	6/45 OD; no light perception OS	Bilateral optic disc swelling; bilateral xeropthalmia with Bitot's spots and severe punctate epithelial erosion	Vitamin A, vitamin B2, vitamin B9, vitamin B12, vitamin D supplementation, maintenance multivitamin and calcium supplement	6/6 OD; 6/18 OS
25	Tarhan et al.^20^	12	F	Vitamin A, vitamin B1, vitamin B9, vitamin B12, vitamin C, vitamin E, zinc, copper	Loss of appetite in the setting of modafinil therapy for narcolepsy, restricted diet	Not specified	Bilateral optic disc edema; bilateral keratinized conjunctiva, Bitot's spots, severe xerophthalmia	Vitamin A, vitamin B1, vitamin B9, vitamin E, zinc supplementation, daily multivitamin, iron, vitamin D, methylprednisolone	“Improved in both eyes”

EBV, Epstein-Barr virus; G-tube, gastrostomy tube; IM, intramuscular; IV, intravenous; MOG, myelin oligodendrocyte glycoprotein; OD, right eye; OS, left eye; PH, pin hole; VA, visual acuity.

## 5 Conclusions

In the developed world, the majority of case reports on nutritional deficiencies causing ocular disease in pediatric patients have focused on children with autism or intellectual disabilities.^5^ Our case series presents three additional pediatric patients found to have nutritional deficiency optic neuropathy without a diagnosis of autism. Patients 1 and 2 were formally diagnosed with ARFID; additionally, patients 1 and 3 had multiple food allergies. In the case of patients 1 and 3, who presented first for declining vision, visual acuity remained stable at the 20/50 to 20/60 range despite nutritional repletion. While the array of nutritional deficiencies varied across patients, low B12 was present in all three. B12 is commonly found in animal products such as meat, poultry, eggs, seafood, and dairy; however, certain processed foods such as chicken nuggets may lack the nutrient entirely.

Nutritional optic neuropathy can be a particularly challenging diagnosis in the pediatric context due to insidious losses in visual acuity, young patients being less aware and/or vocal about changes in vision, lack of obvious physical signs without a dilated fundus exam, and patient hesitancy to communicate with adult providers. In instances where pediatric patients present with optic neuropathy from an unknown cause, a thorough diet and allergy history, as well as nutritional labs, can provide important diagnostic clues. Preliminary history taking should include evaluation of any food allergies or aversions, particularly animal products, and what the patient typically eats and drinks over the course of a day. Initial lab workup should include vitamins A, B1, B6, B12, and copper. Finally, ophthalmologists can initiate prompt multidisciplinary care with gastroenterology, nutrition, and/or psychiatry providers to develop long-term treatment plans which are crucial for prevention of further vision loss and potential for visual recovery.

## CRediT authorship contribution statement

**Rhea W. Teng:** Writing – original draft, Formal analysis, Data curation. **Gena Heidary:** Writing – review & editing. **Ryan A. Gise:** Writing – review & editing, Visualization, Supervision, Conceptualization.

## Patient consent

Written consent to publish this case has not been obtained. This report does not contain any personal identifying information.

## Authorship

All authors attest that they meet the current ICMJE criteria for Authorship.

## Funding

No funding or grant support

## Declaration of competing interest

The authors declare that they have no known competing financial interests or personal relationships that could have appeared to influence the work reported in this paper.
